# Supplementary material for: Deciphering of SOX9 Functions in Pancreatic Cancer Cells
Source: Int J Mol Sci. 2025 Mar 15;26(6):2652. doi: 10.3390/ijms26062652 (PMC11941869; doi:10.3390/ijms26062652)
Supplement: Supplementary file 1 [file ijms-26-02652-s001.zip › Figures S1-S4.pptx]

## Slide 1
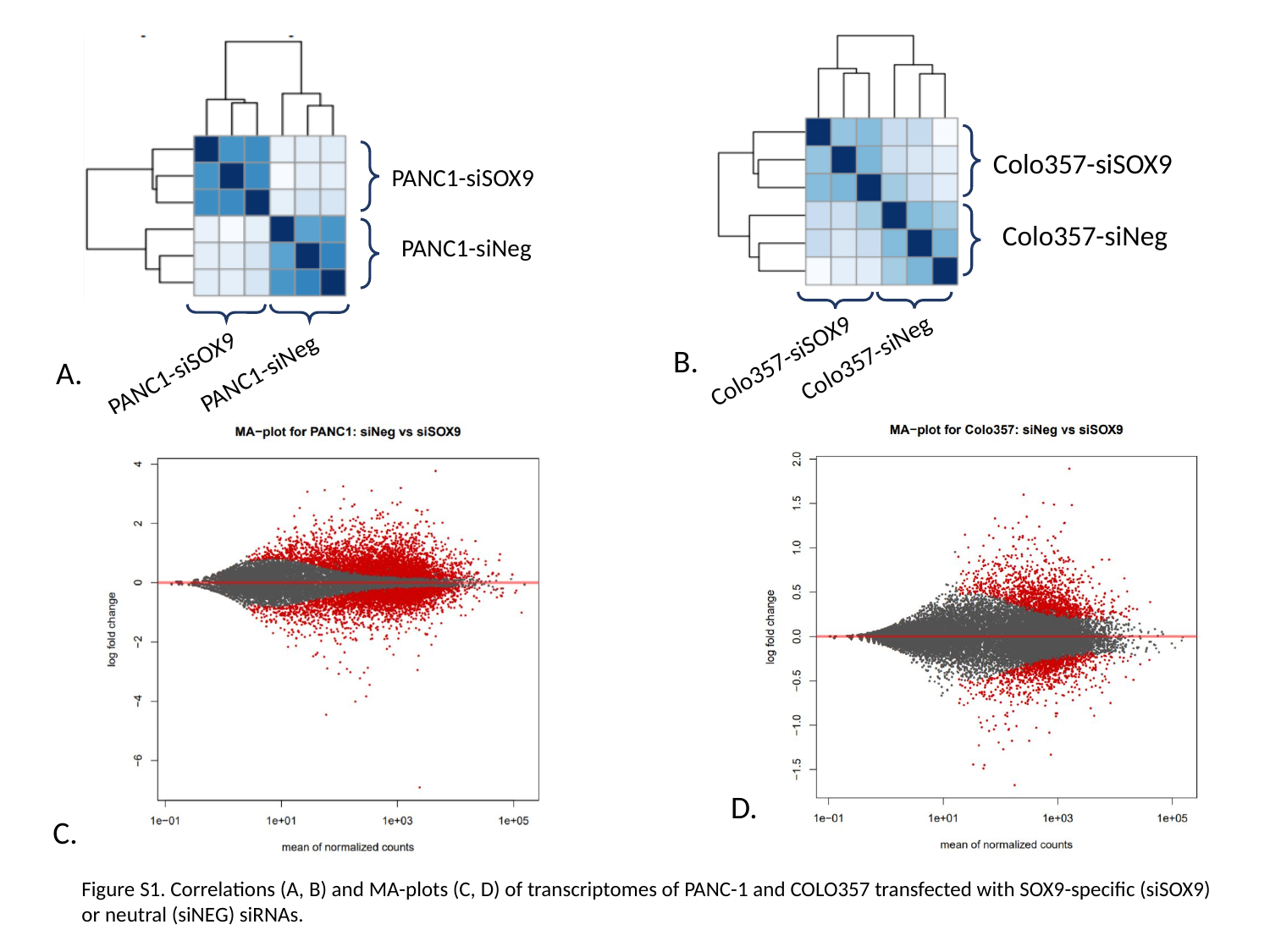

Colo357-siSOX9
Colo357-siNeg
Colo357-siNeg
Colo357-siSOX9
PANC1-siSOX9
PANC1-siNeg
PANC1-siNeg
PANC1-siSOX9
B.
A.
D.
C.
Figure S1. Correlations (A, B) and MA-plots (C, D) of transcriptomes of PANC-1 and COLO357 transfected with SOX9-specific (siSOX9) or neutral (siNEG) siRNAs.

## Slide 2
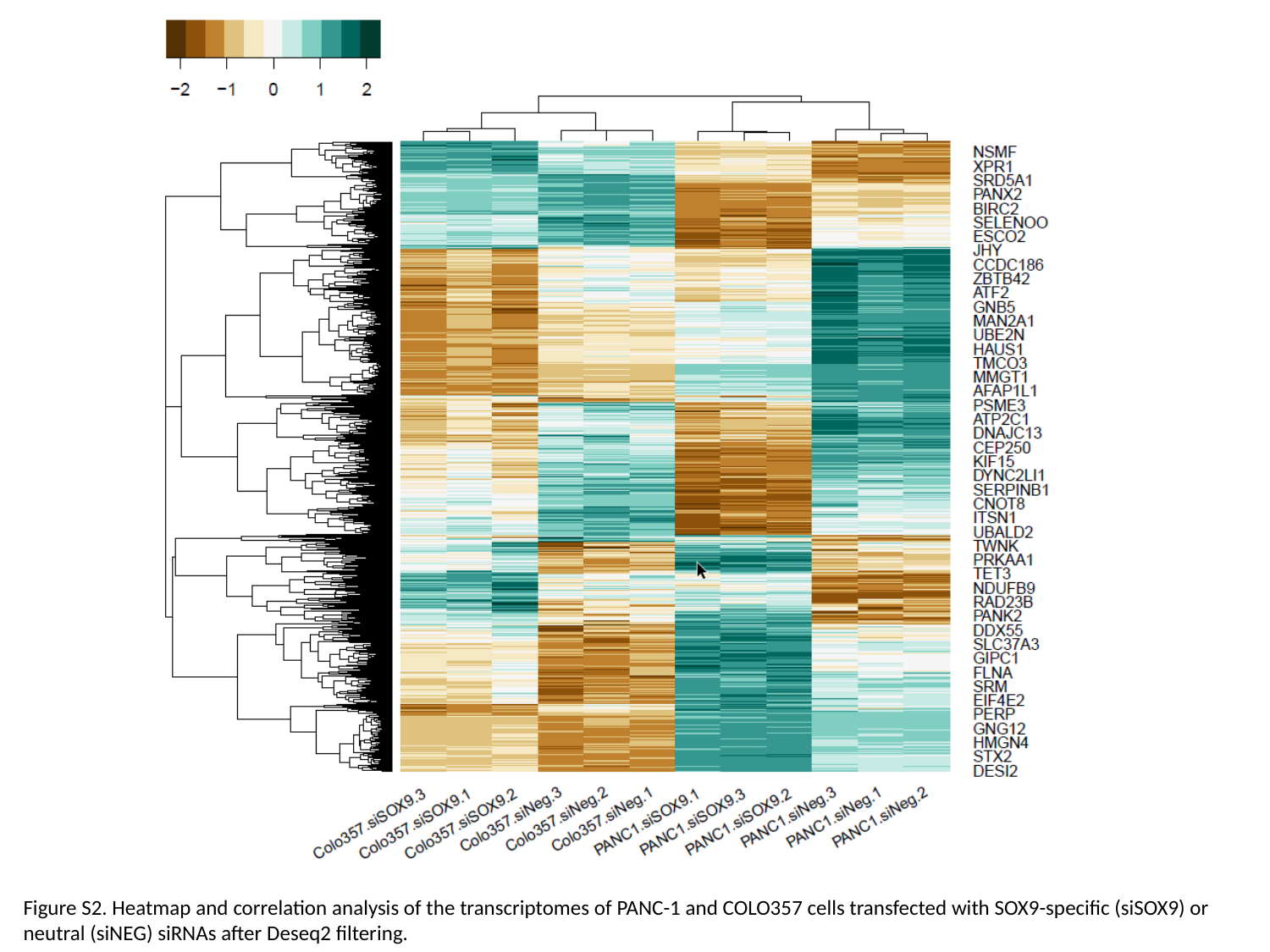

Figure S2. Heatmap and correlation analysis of the transcriptomes of PANC-1 and COLO357 cells transfected with SOX9-specific (siSOX9) or neutral (siNEG) siRNAs after Deseq2 filtering.

## Slide 3
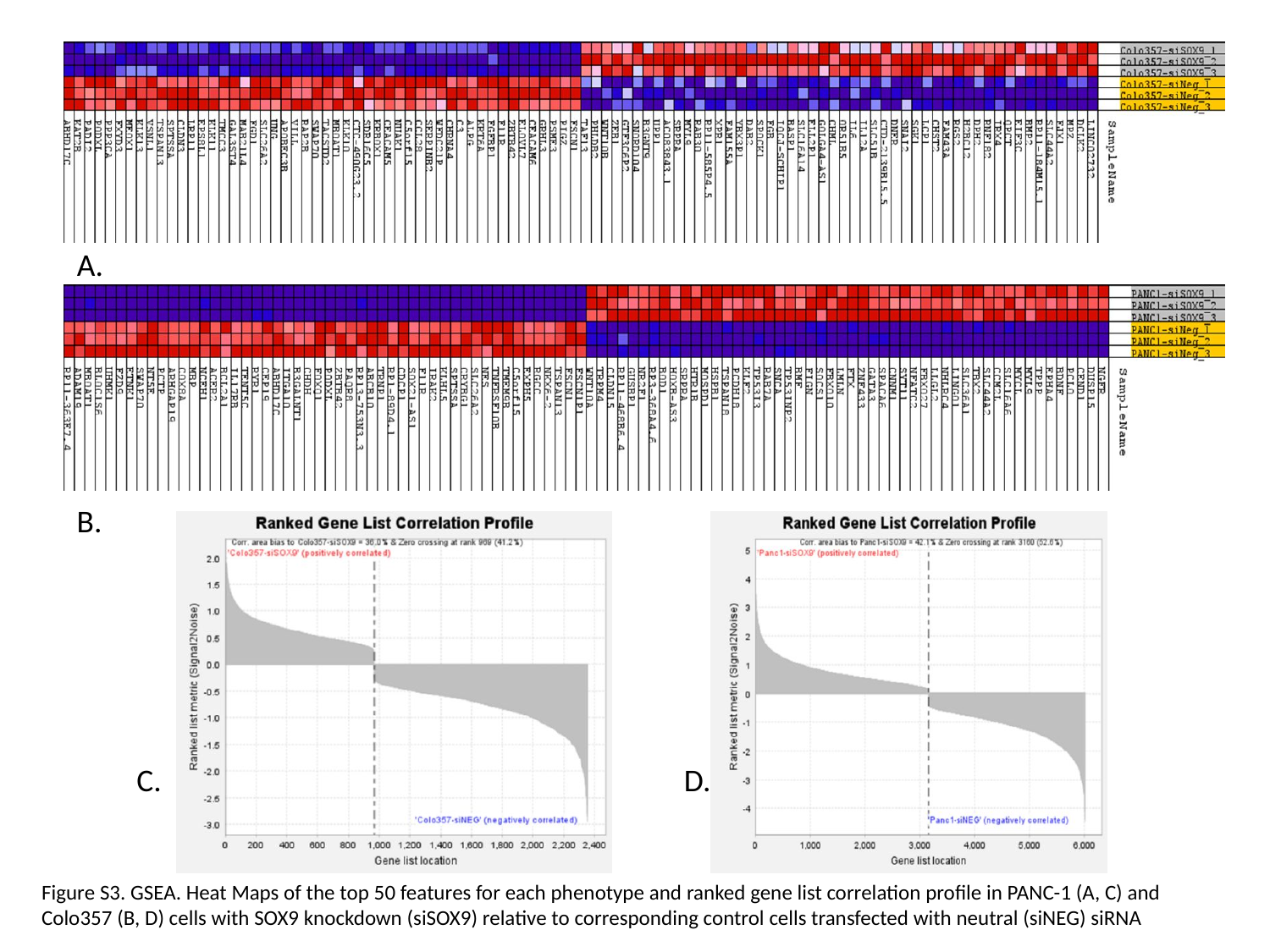

A.
B.
C.
D.
Figure S3. GSEA. Heat Maps of the top 50 features for each phenotype and ranked gene list correlation profile in PANC-1 (A, C) and Colo357 (B, D) cells with SOX9 knockdown (siSOX9) relative to corresponding control cells transfected with neutral (siNEG) siRNA

## Slide 4
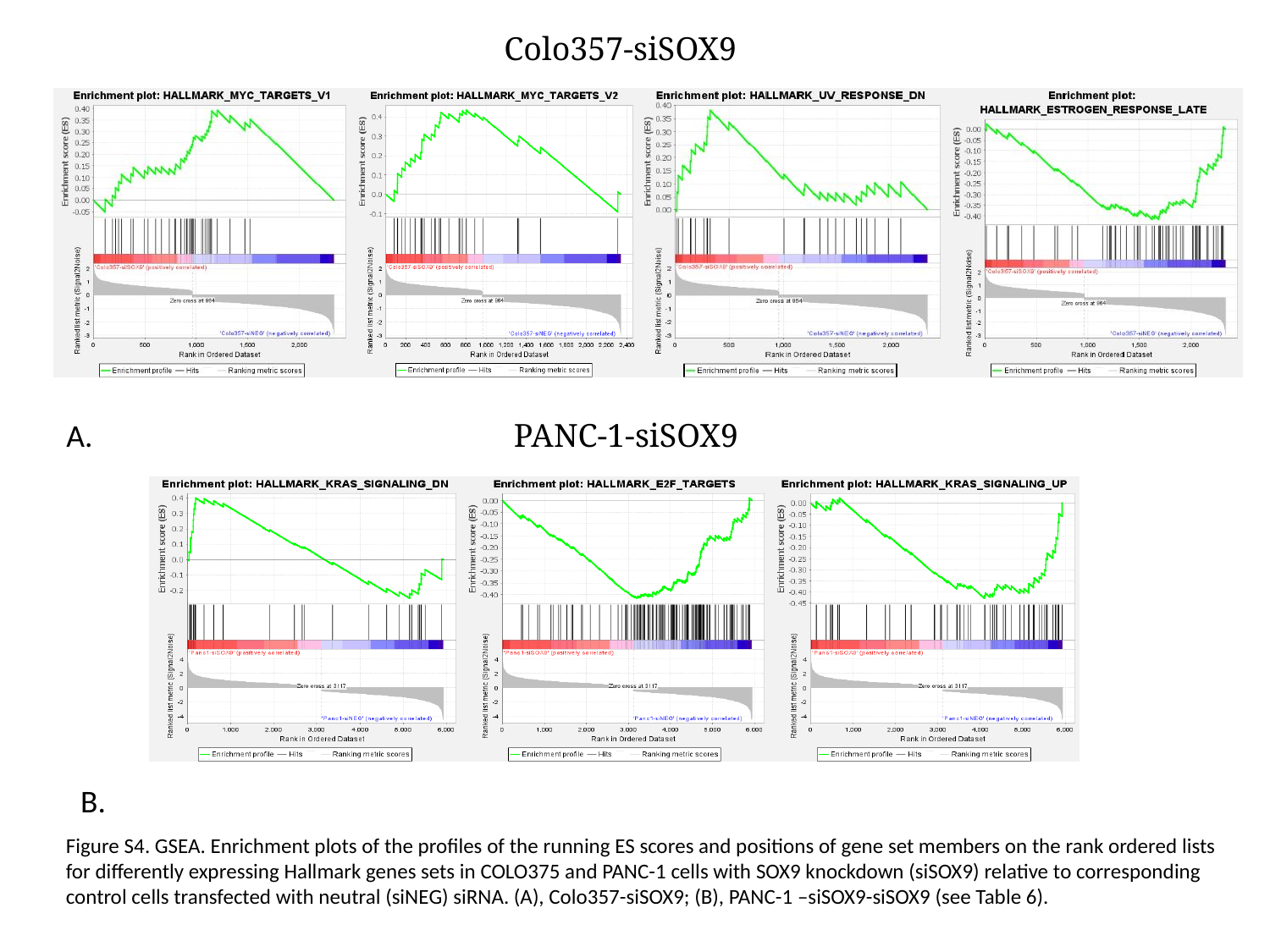

Colo357-siSOX9
A.
PANC-1-siSOX9
B.
Figure S4. GSEA. Enrichment plots of the profiles of the running ES scores and positions of gene set members on the rank ordered lists for differently expressing Hallmark genes sets in COLO375 and PANC-1 cells with SOX9 knockdown (siSOX9) relative to corresponding control cells transfected with neutral (siNEG) siRNA. (A), Colo357-siSOX9; (B), PANC-1 –siSOX9-siSOX9 (see Table 6).
