# Supplementary material for: Deciphering of SOX9 Functions in Pancreatic Cancer Cells
Source: Int J Mol Sci. 2025 Mar 15;26(6):2652. doi: 10.3390/ijms26062652 (PMC11941869; doi:10.3390/ijms26062652)
Supplement: Supplementary file 1 [file ijms-26-02652-s001.zip › Tables S1-S4.pdf]

**Table S1.** List of the antibodies used for Western Blots and immunofluorescence imaging.

Primary antibodies

| **Name** | **Company** | **Host** | **Dilution** |
|---|---|---|---|
| Anti-SOX9 (D8G8H) | Cell Signaling Technology, USA | Rabbit | 1:1000 |
| Anti-GAPDH (0411) | Santa Cruz Biotechnology, USA | Mouse | 1:2500 |

Secondary antibodies

| **Name** | **Company** | **Host** | **Dilution** |
|---|---|---|---|
| Anti-rabbit IgG, HRP-linked | Cell Signaling Technology, USA | Goat | 1:1000 |
| Anti-mouse IgG, HRP-linked | Santa Cruz Biotechnology, USA | Horse | 1:1000 |

**Table S2.** List of the siRNAs used for targeting human SOX9 RNA.

| **siRNA** | **Target** | **Sense Sequence (5' to 3')** |
|---|---|---|
| si#18 | 5'UTR | 5'-GCUCGAAACTGACUGGAAAdTdT-3' |
| si#1299 | ORF | 5'-UCGAGACCUUCGAUGUCAAdTdT-3' |
| si#2691 | 3'UTR | 5'-GAAGCAUUUGGUAAGCUUUdTdT-3' |

**Table S3.** Number of RNA-seq reads by Illumina NovaSeq 6000

| **Name** | **Number of reads** |
|---|---|
| Panc1-siNeg#1 | 18 285 490 |
| Panc1-siNeg#2 | 21 389 266 |
| Panc1-siNeg#3 | 20 095 896 |
| Panc1-siSOX9#1 | 25 895 784 |
| Panc1-siSOX9#2 | 17 024 955 |
| Panc1-siSOX9#3 | 18 858 408 |
| Colo357-siNeg#1 | 19 819 676 |
| Colo357-siNeg#2 | 24 533 581 |
| Colo357-siNeg#3 | 20 071 627 |
| Colo357-siSOX9#1 | 25 904 342 |
| Colo357-siSOX9#2 | 21 230 472 |
| Colo357-siSOX9#3 | 22 180 144 |

**Table S4.** List of the primers used for RT-qPCR.

| Gene | Primer sequence (5' to 3') | PCR product |
|---|---|---|
| SOX9 | FW: 5'- AGCTCTGGAGACTTCTGAACGA -3'<br>RW: 5'- TAGCTGCCCGTGTAGGTGAC -3' | 535 bp |
| HPRT | FW: 5'- GCTATAAATTCTTTGCTGACCTGCTG -3'<br>RW: 5'- AATTACTTTTATGTCCCCTGTTGACTGG -3' | 140 bp |
